# Female versus male migraine: an event-related potential study of visual neurocognitive processing

**DOI:** 10.1186/s10194-019-0995-y

**Published:** 2019-04-23

**Authors:** Yunliang Guo, Song Xu, Shanjing Nie, Mimi Han, Yue Zhang, Jian Chen, Xunyao Hou, Yan Hong, Xueping Liu

**Affiliations:** 1 Department of Senile Neurology, Shandong Provincial Hospital Affiliated to Shandong University, Jinan, 250021 Shandong People’s Republic of China; 2 Anti-Aging Monitoring Laboratory, Shandong Provincial Hospital Affiliated to Shandong University, Jinan, 250021 Shandong People’s Republic of China; 3 Department of Anti-Aging, Shandong Provincial Hospital Affiliated to Shandong University, Jinan, 250021 Shandong People’s Republic of China

**Keywords:** Migraine, Gender-related differences, Event-related potential, P3, Attentive visual processing

## Abstract

**Background:**

Several studies have suggested cognitive deficits in migraineurs, and sex differences have also been observed in migraine, such as a higher prevalence in females. Nevertheless, little is known about gender-related differences in cognitive processing. In this study, we aimed to investigate the effect of gender on neurocognitive processing in migraineurs.

**Methods:**

Altogether, 46 migraine patients without aura (23 females; mean age 32.848 years) during the interictal period and 46 age-matched healthy controls (23 females; mean age 32.652 years) were recruited. The emotional characteristics of participants were evaluated, and attentive processing was analyzed via event-related potential examinations using a three-stimulus visual oddball paradigm.

**Results:**

We found that migraineurs suffered from emotional and visual cognitive processing abnormalities compared with healthy controls, including higher levels of anxiety and reduced P3 amplitude. These parameters were modulated by gender in migraine patients, but not in healthy participants. Our findings indicated that female patients seemed to be more anxious and have more severe impairment in attentive processing of visual stimuli than their male counterparts. The gender-related differences in migraineurs were further validated using event-related potential difference waveforms.

**Conclusions:**

These results suggested that migraine might have an additional influence on females and lead to more dysfunction in their interictal neurocognitive processing. Our findings provide evidence that a gender effect exists in migraineurs, which should be considered when designing experiments and exploring treatment approaches. The gender-related differences and underlying mechanisms deserve further investigation for patients with migraine.

**Electronic supplementary material:**

The online version of this article (10.1186/s10194-019-0995-y) contains supplementary material, which is available to authorized users.

## Background

Migraine is a debilitating pain disorder, characterized by severe throbbing headaches, photophobia, phonophobia and gastrointestinal disturbances, which causes lower quality of life, impaired sociability, loss productivity and even disability [1]. Psychiatric disorders, such as anxiety and depression, are common comorbidities of migraine [2], and patients with chronic migraine often exhibit affective temperamental dysregulation and suicidal behaviors as well [3]. These specific psychological characteristics may significantly contribute to the psychosocial impairment and negatively modify the outcomes of migraineurs. In addition, some studies have indicated that migraineurs could suffer from cognitive deficits in several domains [4, 5] or subsequent dementia [6]. Migraine is closely associated with an increased prevalence of gray matter volume reduction in frontal cortex and cingulate gyrus, deep white matter lesions and subclinical cerebral infarcts as well [7, 8], which can all lead to cognitive decline [9].

Interestingly, there have been evidence indicating the existence of gender differences in migraine, including: 1) the prevalence is about twice as high in females compared with males [10], 2) most associated symptoms are more prevalent and severe in women [11], 3) female migraineurs have a greater number of comorbid diseases and are more likely to suffer from psychiatric comorbidities [12, 13], 4) higher headache-related disability and healthcare resource utilization in females [14], 5) gender-related responses to anti-migraine treatments such as triptans [15], 6) the potential influence of estrogen fluctuations in female migraineurs [16], and 7) sex differences in structural and functional brain alterations [17, 18]. Moreover, gender is also an important influential element in cognitive-behavioral performance [19, 20]. Nevertheless, to date, there has been little discussion on cognitive differences between male and female patients with migraine. Owing to difference in the incidence of this disease, there is the high possibility that migraine might exert diverse effects on cognitive processing between males and females.

Event-related potential (ERP) is a relatively objective and noninvasive neurophysiological examination that can reflect underlying brain activities during cognitive processing, and has been increasingly employed as a cognitive marker in various neurological disorders. P3 (also called P300), the most investigated ERP component, is regarded as an effective index of cerebral information processing [21].

The aim of the present study was to characterize the effect of gender on migraine and possible interactions between gender and the disease in neurocognitive processing using a modified visual oddball paradigm. We hypothesized that migraineurs overall suffered from attentive processing impairment and attentional ERP abnormalities, such as reduced P3 amplitude and/or prolonged P3 latency. More importantly, considering the reported gender-related discrepancies in associated symptoms, comorbidities, headache-related disability and cerebral dysfunction [11, 13, 14, 17], the impairment severity might also not be the same between male and female migraineurs. The attentive visual processing in migraine sufferers, assessed by corresponding original/difference ERP components, might map onto observed gender-related functional differences.

## Methods

### Participants and criteria

Altogether, we recruited 49 outpatients with migraine without aura (25 females) from Shandong Provincial Hospital Affiliated to Shandong University. Besides, 48 age-matched healthy controls (24 females) recruited from hospital/ laboratory staff participated in this study. They did not suffer from any recurrent headache or drug/alcohol abuse.

All patients underwent necessary neurological examinations by two specialized neurologists (XL and XH), and were diagnosed according to the beta version of the International Classification of Headache Disorders, 3rd edition (ICHD-3 beta). Migraineurs were in the interictal period when enrolled, and had no attack 72 h prior to the recording and no symptoms of developing a migraine during or 72 h after the recording. The inclusion criteria for patients were: 1) fulfilling the criteria for migraine without aura as classified in the ICHD-3 beta, 2) aged between 18 and 45 years, 3) at least one migraine episode per month and at least 1 year’s migraine history, and 4) outside migraine attacks during the recording, as well as at least 72 h before and after the experiment. The exclusion criteria for patients were: 1) receiving prophylactic anti-migraine therapy, 2) a history of analgesic drug overuse or addiction, 3) a drug/substance abuse or dependency, 4) general neurological disorders, 5) a history of mixed headache types, and 6) abnormal findings on neurological examinations or brain morphology indicating other potential neurological diseases. We also excluded participants who were illiterate, vision impaired, suffering from depression, or having suicide ideation and/or previous suicide attempts. All female subjects were verified to take no oral contraceptives for at least 1 week.

Within the study, migraine characteristics were obtained by a standardized interview using a structured questionnaire, including: 1) the frequency and duration of attacks during the previous year, 2) the history of migraine, and 3) the headache score representing a rating of the most severe migraine suffered over the past year by visual analog scale (VAS), with 0 indicating no pain and 10 worst possible pain respectively.

Furthermore, 3 migraineurs (2 females) were excluded – two for excessive artefacts (blink and electromyographic activity) within electroencephalogram (EEG) data and one for incomplete clinical characteristics. So 46 patients (23 females) in total were included. In terms of controls, 2 participants (1 female) had to be excluded. One for lack of emotional rating scales and the other for technical problems during recording. Thus, we finally included 46 controls (23 females) for further analysis.

### Emotional evaluation

In this study, we used Self-Rating Anxiety Scale (SAS) and Self-Rating Depression Scale (SDS) to evaluate emotional state of subjects as described in our previous study [22]. In brief, all participants responded to a series of questions associated with daily life to determine whether they had emotional abnormalities and severity. Each scale contains 20 items rated on a four-level Likert scale, ranging from “never occurring or just a little of the time” to “most of the time” [23, 24]. Responses were summed to obtain a total score, and higher scores suggested greater levels of anxiety or depression symptomatology. The anxiety or depression state was defined as score of SAS above 49 or score of SDS above 52, respectively.

### Stimuli and procedure

All subjects were seated comfortably in an armchair in an electrical shielded and sound attenuated chamber, and were directed to fixate and attend to a fixation cross at the center of a screen (23 in.) situated 0.5 m in front of them. A modified visual oddball paradigm was applied, and 501 stimuli divided into three separate blocks were presented in a random fashion with each stimulus lasting for 400 ms and a fixed inter-stimulus interval of 400 ms. Stimuli were comprised of standard (smaller circle, *n* = 381, *p* = 0.76), target (larger circle, *n* = 60, *p* = 0.12) and novel (square, *n* = 60, *p* = 0.12) stimuli. During the experiment, participants were instructed to discriminate target stimuli (*n* = 60, 20 for every block) from other stimuli as accurately and quickly as possible, and were required to count the number of target stimuli presented in each block mentally. Thereafter, the number was reported, followed by calculation and recording of accuracy based on counting mistakes. Subjects with accuracy below 90% were not considered in data analysis.

### Recording of EEG signals

The continuous EEG signals (sampling rate 1000 Hz, low pass filter 100 Hz) were obtained from midline Fz, Cz and Pz electrode sites in accordance with the 10–20 international system using Neurolab EEG/ERPs 32 Channel Amplifier. All scalp electrodes were referenced to left mastoid signals, with right mastoid as ordinary recording site. Electrooculogram (EOG) was recorded with electrodes placed above and below the right eye and 10 mm from the outer canthi. Electrode impedance was maintained below 5 kΩ throughout the experiment.

### EEG data analysis and measurement

ASA 4.9.3 software was used for off-line analysis of EEG data. The data were re-referenced to the average of bi-mastoid signals. EOG artifacts were removed using independent component analysis (ICA) as suggested by Jung et al. [25], followed by bandpass filtering at 0.1–30 Hz (24 dB/octave). Afterwards, EEG signals obtained were segmented into the epoch from 200 ms pre-stimulus to 800 ms post-stimulus, and a − 200 to 0 ms pre-stimulus baseline was applied for all ERP waveform corrections. Trials contaminated by high-frequency noise, other extra-cerebral artifacts, or peak-to-peak deflection exceeding ±100 μV amplitude at any electrode were automatically excluded from averaging. The segments were averaged separately for standard, target and novel stimuli, and grand averaged waveforms were generated using single-subject waveforms for further analysis.

**Fig. 1:**
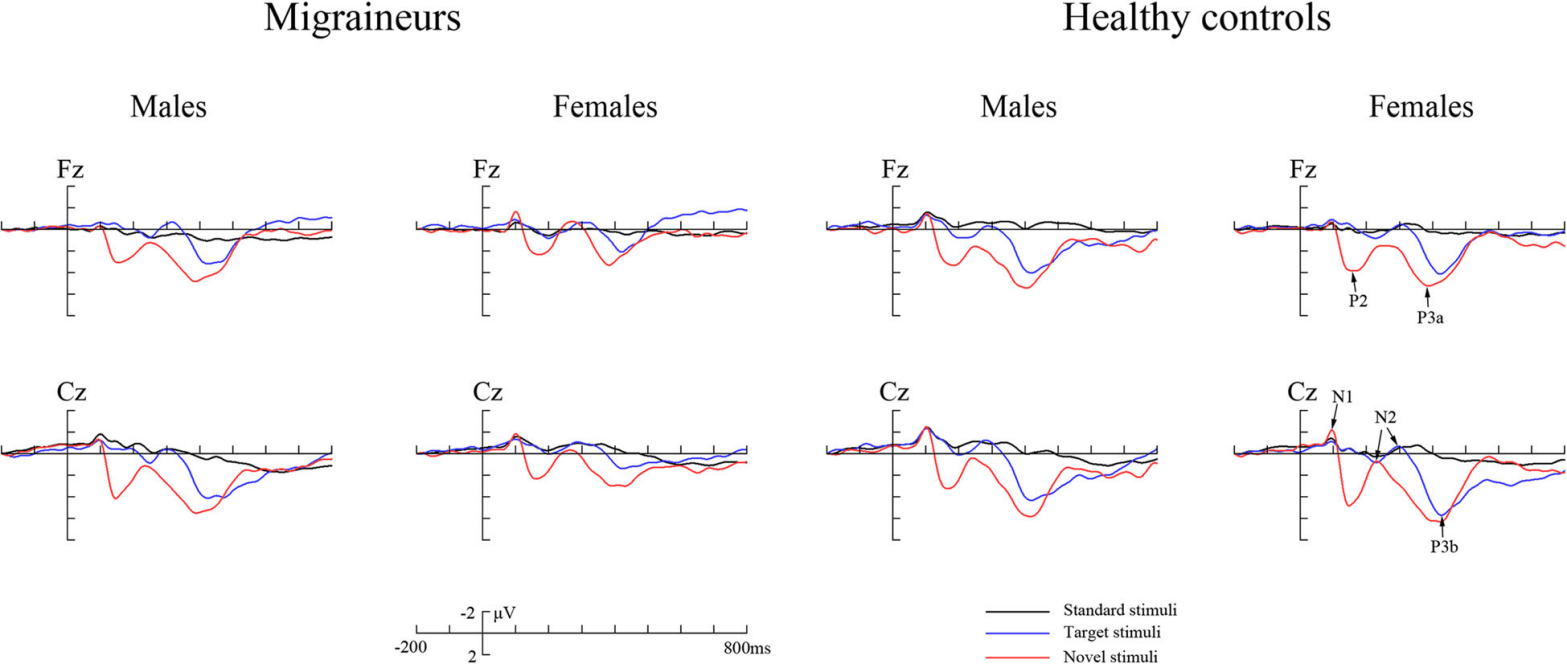
The grand averaged ERP waveforms in migraineurs and healthy controls at Fz and Cz, respectively. Original waveforms are elicited by standard (black), target (blue) and novel stimuli (red line)

The peak amplitudes and latencies of original ERP components were measured relative to the pre-stimulus baseline period. The positive peak between 300 and 500 ms post-stimulus, the negative peak between 50 and 190 ms, the positive peak between 110 and 270 ms, and the negative peak between 210 and 370 ms were applied to define the P3, N1, P2 and N2 components, respectively (Fig. 1).

To reliably observe and better assess the target and novel effects, the difference waveforms were obtained by subtracting ERPs in response to standard stimuli from those in response to target and novel stimuli, respectively, and the components which showed significant gender difference in migraine patients were further analyzed at corresponding time intervals in difference waveforms (see Fig. 3).

The experimenters were blind regarding the subject’s diagnosis and gender throughout preprocessing and measurements of EEG data.

### Statistical analysis

Quantitative data were presented as mean ± standard deviation (SD). To compare demographic features, emotional characteristics and behavioral parameters, a two-way analysis of variance (ANOVA) was conducted. Student’s *t*-test for independent samples (two-tailed) was used for comparisons of clinical features between male and female patients. Additionally, amplitudes and latencies of original ERP components were analyzed by repeated-measures ANOVA, with stimulus (standard, target and novel) and electrode (Fz, Cz and Pz) as within-subject factors, while with group (migraineurs vs. healthy controls) and gender (male vs. female) as between-subject factors. For difference ERP components, repeated-measures ANOVA was performed with electrode (Fz, Cz and Pz) as a within-subject factor, while with group (migraineurs vs. healthy controls) and gender (male vs. female) as between-subject factors. The degrees of freedom were corrected for nonsphericity using Greenhouse–Geisser epsilon if necessary. Further post-hoc analysis using Bonferroni correction was conducted for significant results. Statistical calculations were carried out with SPSS 23.0 (SPSS Inc., Chicago, IL, USA). The significance level was fixed at 0.05 for all analyses, and effect sizes were reported as partial eta squared (*η*^2^).

## Results

### Subject characteristics

Tables 1 and 2 show demographic and clinical characteristics. We did not find effects or interactions in age and education level of participants (*p*s > 0.3). There existed no significant sex differences in migraine frequency (*p =* 0.763), duration of attacks (*p =* 0.897), history of migraine (*p =* 0.798) and headache score (*p =* 0.658) either.

**Table 1:** Subject characteristics

	Migraineurs	Healthy controls
N	46	46
Age, years	32.848 ± 6.467	32.652 ± 6.360
Age range, years	20–45	21–45
Education, years	13.565 ± 2.802	14.022 ± 4.726
Migraine frequency, times per month	3.783 ± 2.890	–
Duration of migraine, hours	20.696 ± 20.069	–
History of migraine, years	7.630 ± 5.097	–
Headache score, 0–10	5.457 ± 0.982	–
SAS score	42.913 ± 8.453	35.543 ± 6.217
SDS score	40.043 ± 9.716	36.522 ± 7.580

Data were expressed as mean ± SD

*SAS* Self-Rating Anxiety Scale, *SDS* Self-Rating Depression Scale

**Table 2:** Subject characteristics

	Migraineurs		Healthy controls	
	Males	Females	Males	Females
N	23	23	23	23
Age, years	32.957 ± 6.335	32.739 ± 6.737	32.870 ± 5.802	32.435 ± 6.999
Age range, years	21–43	20–45	21–45	22–45
Education, years	13.217 ± 2.923	13.913 ± 2.695	13.652 ± 4.052	14.391 ± 5.383
Migraine frequency, times per month	3.652 ± 3.024	3.913 ± 2.811	–	–
Duration of migraine, hours	20.304 ± 20.195	21.087 ± 20.387	–	–
History of migraine, years	7.435 ± 4.143	7.826 ± 5.992	–	–
Headache score, 0–10	5.391 ± 0.941	5.522 ± 1.039	–	–
SAS score	40.217 ± 7.323	45.609 ± 8.794	35.870 ± 6.398	35.217 ± 6.157
SDS score	39.000 ± 9.313	41.087 ± 10.202	36.130 ± 7.092	36.913 ± 8.179

Data were expressed as mean ± SD

*SAS* Self-Rating Anxiety Scale, *SDS* Self-Rating Depression Scale

### Emotional features

The emotional evaluation indicated that migraine patients were more anxious than healthy controls (*F*(1,88) = 23.814, *p* < 0.001, partial *η*^2^ = 0.213), while across groups, SAS score was not affected by gender (*F*(1,88) = 2.462, *p* = 0.120). We found significant group × gender interaction (*F*(1,88) = 4.004, *p* = 0.048), and both male and female migraineurs tended to be more anxious than their control counterparts (*F*(1,88) = 4.144, *p* = 0.045 for males and *F*(1,88) = 23.674, *p* < 0.001 for females). In addition, the SAS score was higher in female patients compared with male patients (*F*(1,88) = 6.373, *p* = 0.013), but not for healthy participants (*F*(1,88) = 0.093, *p =* 0.761) (see Table 2).

No group, gender effects or group × gender interaction was obtained in SDS score (*p*s > 0.05).

### Behavioral data

For accuracy of subjects (male migraineurs, 97.2%; female migraineurs, 97.1%; male controls, 97.6%; female controls, 97.9%), no effects or interaction was observed significant (*p*s > 0.2).

### Original ERP analysis

The grand averaged ERP waveforms are demonstrated in Fig. 1, and analyses of multiple original ERP components are summarized in Table 3.

**Table 3:** Results and analyses of original ERP components

		Migraineurs		Healthy controls		Statistics		
Original ERPs		Males	Females	Males	Females	Group *F*(1,88)	Gender *F*(1,88)	Group × Gender *F*(1,88)
P3	Amplitude (μV)	4.321 ± 3.390	2.848 ± 2.613	4.451 ± 3.504	4.911 ± 4.025	12.564**	2.684	9.757**
	Latency (ms)	407.130 ± 39.851	404.942 ± 38.794	408.531 ± 37.379	411.034 ± 38.426	1.465	0.003	0.574
N1	Amplitude (μV)	−1.777 ± 1.669	− 2.436 ± 1.991	−2.656 ± 2.267	−2.131 ± 1.951	1.042	0.056	4.434*
	Latency (ms)	107.106 ± 30.395	112.700 ± 30.348	112.058 ± 30.020	111.097 ± 33.639	0.212	0.406	0.812
P2	Amplitude (μV)	2.926 ± 2.128	1.915 ± 1.588	2.551 ± 2.366	3.084 ± 3.041	3.043	1.101	11.518**
	Latency (ms)	196.391 ± 39.526	193.541 ± 36.700	192.966 ± 35.580	190.720 ± 38.724	0.560	0.373	0.005
N2	Amplitude (μV)	−0.864 ± 2.284	−1.511 ± 1.868	−1.261 ± 2.380	−0.919 ± 2.590	0.104	0.254	2.676
	Latency (ms)	281.449 ± 38.000	287.420 ± 32.518	287.444 ± 36.686	283.198 ± 35.468	0.066	0.063	2.203

Data were expressed as mean ± SD

*P < 0.05, **P < 0.01 by repeated-measures ANOVA (Bonferroni correction)

#### P3 component

**Fig. 2:**
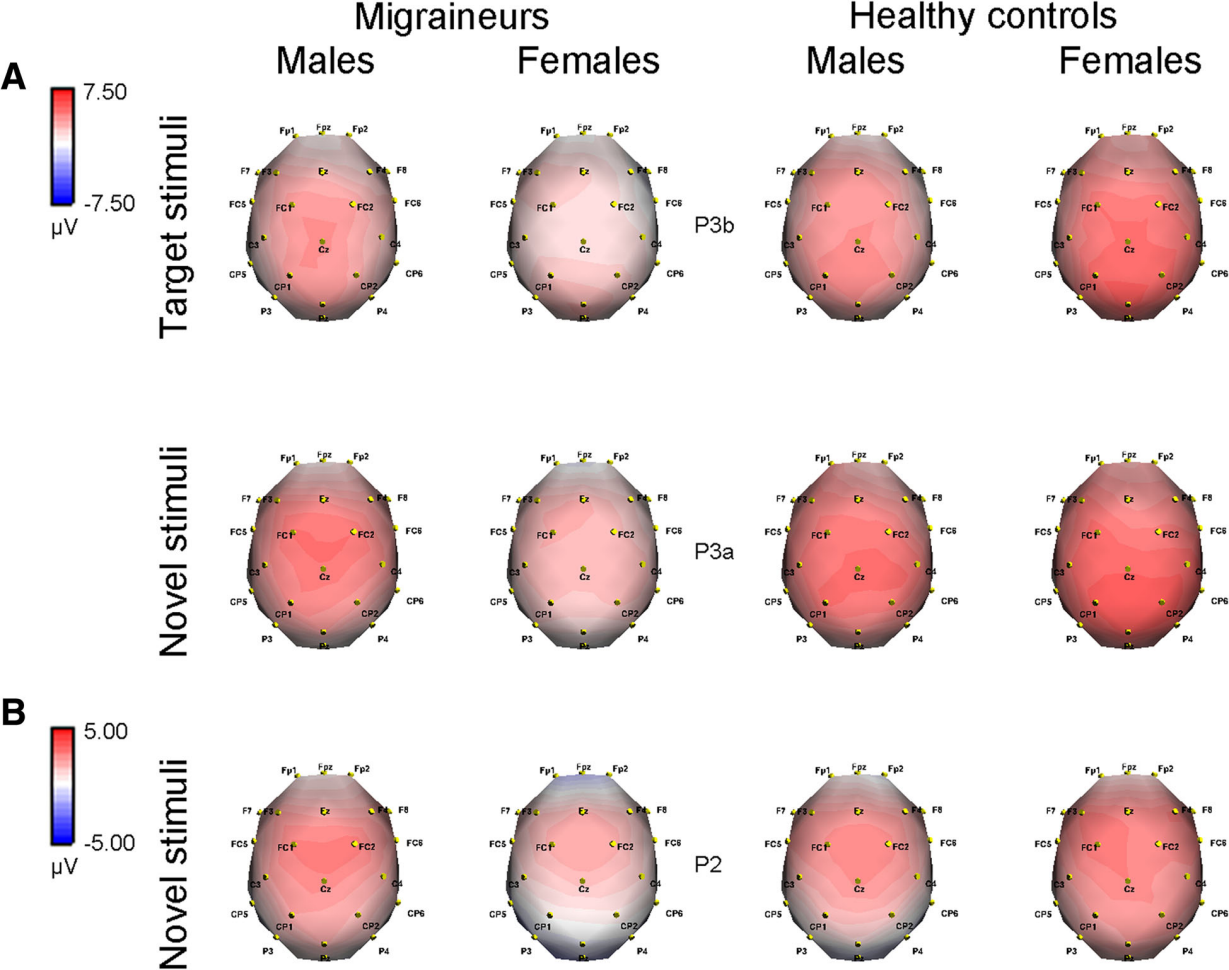
Topographical voltage distributions of original ERP components in migraineurs and healthy controls, respectively. **a** P3 elicited by different stimuli: target stimuli (P3b) at 410–425 ms and novel stimuli (P3a) at 390–415 ms; (**b**) P2 elicited by novel stimuli at 170–185 ms. Red color denotes a positive and blue color a negative potential

Figure 2a depicts topographies of voltage distribution for P3b and P3a components. P3 amplitude showed remarkable main effect of stimulus (*F*(1.722,151.528) = 113.387, *p* < 0.001, partial *η*^2^ = 0.563), with a maximum of 5.790 μV (SD 3.458) for novel stimulus (P3a). The amplitude was markedly reduced in migraine patients (3.584 ± 3.112 μV) compared with healthy controls (4.681 ± 3.776 μV, *p* = 0.001, partial *η*^2^ = 0.125). Although no difference was found between genders (*p* = 0.105), the interaction of group × gender was statistically significant (*p* = 0.002, partial *η*^2^ = 0.100) (Table 3). Post-hoc analysis revealed no noteworthy difference between male migraineurs and male controls (*F*(1,88) = 0.089, *p* = 0.767), but for females, the amplitude was lower in migraineurs (*F*(1,88) = 22.232, *p* < 0.001, partial *η*^2^ = 0.202). Furthermore, P3 amplitude was not modulated by gender in controls (*F*(1,88) = 1.103, *p* = 0.297), while in patients, it was larger for males than for females (*F*(1,88) = 11.338, *p* = 0.001, partial *η*^2^ = 0.114) (Fig. 2a).

As for P3 latency, remarkable main effect of stimulus (*F*(1.975,173.800) = 12.386, *p* < 0.001, partial *η*^2^ = 0.123) was obtained, of which novel-elicited P3a was fastest (398.830 ± 38.123 ms). The analysis did not verify other significant results (*p*s > 0.2).

#### N1 component

Although neither group (*p* = 0.310) nor gender effects (*p* = 0.813) was found significant in N1 amplitude, there existed a noticeable interaction of group × gender (*p* = 0.038). Further post-hoc tests indicated that in males, the amplitude was smaller for migraineurs than for controls (*F*(1,88) = 4.887, *p* = 0.030), while not in females (*F*(1,88) = 0.588, *p* = 0.445). N1 amplitude was not affected by gender in both groups (*p*s > 0.05).

No significant difference was discovered in analysis of N1 latency (*p*s > 0.1).

#### P2 component

In terms of P2 amplitude, significant stimulus effect (*F*(1.277, 112.348) = 113.309, *p* < 0.001, partial *η*^2^ = 0.563) was obtained, and novel stimulus elicited the largest amplitude (4.340 ± 2.728 μV). As shown in Table 3, the group (*p* = 0.085) and gender effects (*p* = 0.297) did not reach significant levels, while there appeared to be noticeable group × gender interaction (*p* = 0.001, partial *η*^2^ = 0.116). Subsequent analysis displayed that in females, the amplitude was reduced in migraineurs compared with controls (*F*(1,88) = 13.201, *p* < 0.001, partial *η*^2^ = 0.130), but this phenomenon did not exist in males (*F*(1,88) = 1.360, *p* = 0.247). No difference was observed between male controls and female controls (*F*(1,88) = 2.749, *p* = 0.101). By contrast, the amplitude was lower in female migraineurs in comparison with male migraineurs (*F*(1,88) = 9.871, *p* = 0.002, partial *η*^2^ = 0.101) (see Fig. 2b for novel-elicited P2).

As for P2 latency, the electrode effect was proved remarkable (*F*(1.768,155.604) = 34.315, *p* < 0.001, partial *η*^2^ = 0.281). Other effects or interactions were not significant (*p*s > 0.4).

#### N2 component

Neither group (*p* = 0.748 for amplitude; *p* = 0.797 for latency) nor gender effects (*p* = 0.615 for amplitude; *p* = 0.803 for latency) was found significant in N2 analysis. No group × gender interactions (*p* = 0.105 for amplitude; *p* = 0.141 for latency) was obtained either.

### Difference ERP analysis

**Fig. 3:**
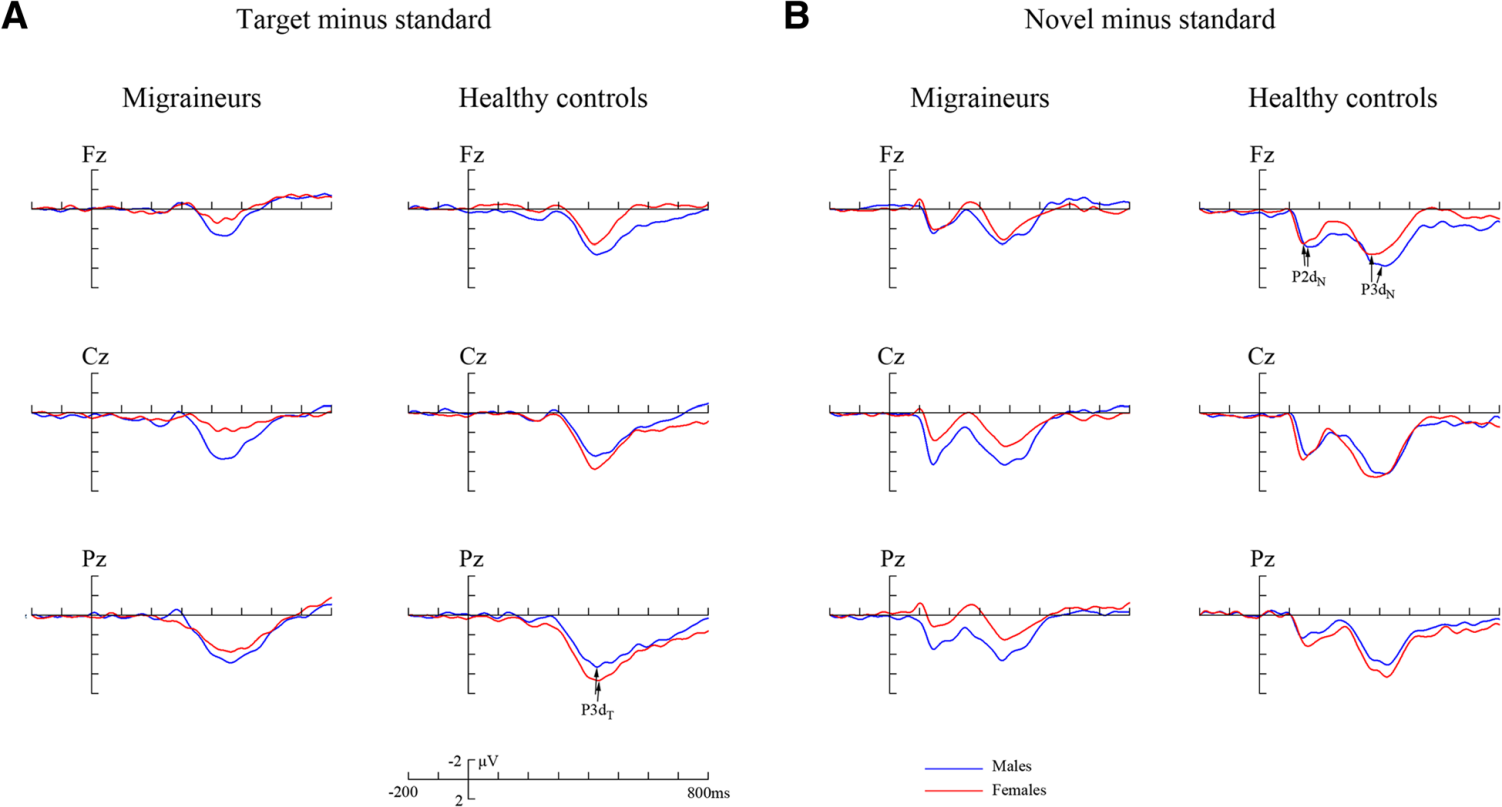
The averaged difference waveforms in migraineurs and healthy controls at Fz, Cz and Pz, respectively. **a** Target minus standard difference ERPs; (**b**) novel minus standard difference ERPs. P3d_T_, P3d_N_ and P2d_N_ represent the P3 target effect, P3 and P2 novel effects, respectively. Blue line represents males and red line females

**Fig. 4:**
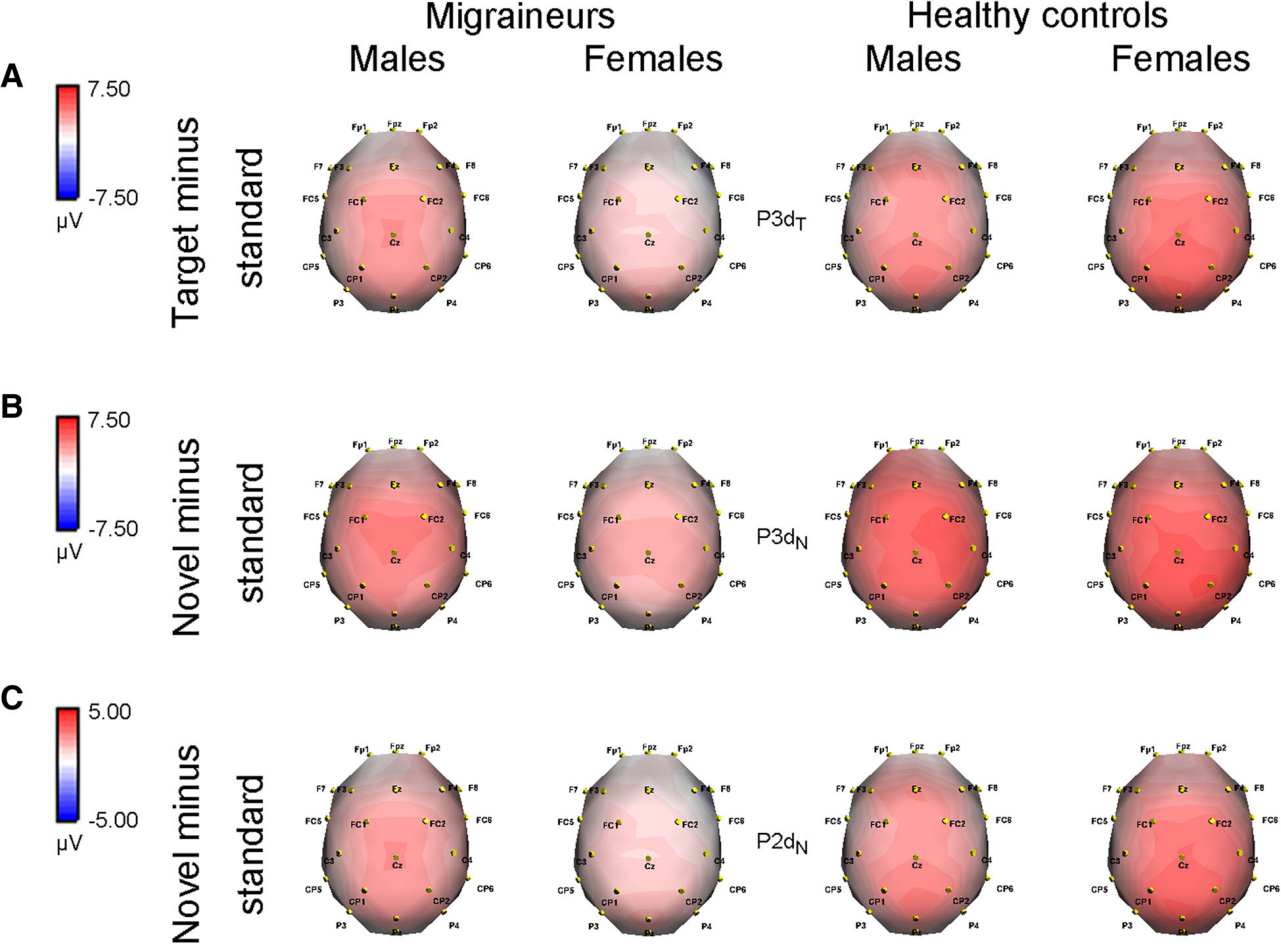
Topographical voltage distributions of difference ERP components in migraineurs and healthy controls, respectively. **a** P3d_T_ at 410–430 ms; (**b**) P3d_N_ at 380–400 ms; (**c**) P2d_N_ at 160–180 ms. P3d_T_, P3d_N_ and P2d_N_ represent the P3 target effect (target minus standard), P3 and P2 novel effects (novel minus standard), respectively. Red color denotes a positive and blue color a negative potential

The difference waveforms are displayed in Fig. 3, in which P3 and P2 difference components were further analyzed (see Table 4 and Fig. 4).

**Table 4:** Results and analyses of difference ERP components

		Migraineurs		Healthy controls		Statistics		
Difference ERPs		Males	Females	Males	Females	Group *F*(1,88)	Gender *F*(1,88)	Group × Gender *F*(1,88)
P3d_T_	Amplitude (μV)	5.061 ± 2.200	3.086 ± 1.668	5.698 ± 3.109	6.367 ± 3.840	20.225***	2.245	9.215**
	Latency (ms)	428.406 ± 34.649	417.841 ± 39.290	423.594 ± 37.222	415.478 ± 36.722	0.407	2.760	0.047
P3d_N_	Amplitude (μV)	5.606 ± 2.007	3.787 ± 1.740	6.288 ± 2.137	6.950 ± 3.440	26.334***	2.383	10.955**
	Latency (ms)	387.754 ± 36.008	395.580 ± 35.748	396.464 ± 32.580	384.058 ± 38.505	0.056	0.148	2.890
P2d_N_	Amplitude (μV)	4.529 ± 2.628	3.072 ± 2.208	4.407 ± 2.283	4.922 ± 2.651	6.643*	1.974	8.650**
	Latency (ms)	165.362 ± 34.496	162.971 ± 32.135	176.594 ± 35.897	170.522 ± 37.271	2.867	0.582	0.110

Data were expressed as mean ± SD

P3d_T_, P3d_N_ and P2d_N_ represent the P3 target effect (target minus standard), P3 and P2 novel effects (novel minus standard), respectively

*P < 0.05, **P < 0.01, ***P < 0.001 by repeated-measures ANOVA (Bonferroni correction)

### P3d_T_ and P3d_N_ components

The analysis of P3d_T_ and P3d_N_ (i.e., the P3 target and novel effects, respectively) amplitudes both demonstrated significant electrode effects (*F*(1.976, 173.868) = 23.436, *p* < 0.001 for P3d_T_; *F*(1.951, 171.712) = 5.170, *p* = 0.007 for P3d_N_). The amplitudes were smaller for migraineurs (P3d_T_: 4.073 ± 2.183 μV, P3d_N_: 4.696 ± 2.082 μV) than for controls (P3d_T_: 6.033 ± 3.497 μV, P3d_N_: 6.619 ± 2.872 μV; *p* < 0.001, partial *η*^2^ = 0.187 for P3d_T_; *p* < 0.001, partial *η*^2^ = 230 for P3d_N_). We failed to find gender effects (*p*s > 0.1), while the significant interactions of group × gender (*p* = 0.003, partial *η*^2^ = 0.095 for P3d_T_; *p* = 0.001, partial *η*^2^ = 0.111 for P3d_N_) were observed (Table 4). Further comparisons suggested that in females, migraineurs exhibited reduced amplitudes (*F*(1,88) = 28.371, *p* < 0.001, partial *η*^2^ = 0.244 for P3d_T_; *F*(1,88) = 35.629, *p* < 0.001, partial *η*^2^ = 0.288 for P3d_N_), but not for males (*p*s > 0.2). Moreover, the amplitudes were remarkably affected by gender in patient group (*F*(1,88) = 10.278, *p* = 0.002, partial *η*^2^ = 0.105 for P3d_T_; *F*(1,88) = 11.778, *p* = 0.001, partial *η*^2^ = 0.118 for P3d_N_), while not in control group (*p*s > 0.2) (Figs. 3a, b, 4a and b).

No statistically significant difference was obtained in latencies of P3d_T_ and P3d_N_ (*p*s > 0.05).

### P2d_N_ component

The P2d_T_ (target minus standard) amplitude of participants did not differ significantly from zero in all electrodes (all *p* > 0.05 by paired *t*-test) (Fig. 3a), thus only P2d_N_ (novel minus standard) was measured and analyzed. Although there was no difference between genders (*p* = 0.164) in P2d_N_ amplitude, the group effect (*p* = 0.012, partial *η*^2^ = 0.070) and group × gender interaction (*p* = 0.004, partial *η*^2^ = 0.089) were observed (see Table 4). Post-hoc tests revealed remarkable differences between female migraineurs and female controls (*F*(1,88) = 15.227, *p* < 0.001, partial *η*^2^ = 0.148), as well as between female migraineurs and male migraineurs (*F*(1,88) = 9.444, *p* = 0.003, partial *η*^2^ = 0.097). Other comparisons did not reach significant levels (*p*s > 0.2) (Figs. 3b and 4c).

We did not discover any notable effects or interactions in P2d_N_ latency (*p*s > 0.05).

## Discussion

In the present study, by using a modified visual oddball paradigm, we discovered emotional and interictal attentive processing abnormalities in migraineurs, including higher levels of anxiety, reduced P3, P3d_T_ and P3d_N_ amplitudes, and lower P2d_N_ amplitudes. Moreover, gender-related differences of these parameters in migraine patients were also observed, and female patients tended to suffer from more severe abnormalities in these domains compared with male patients, such as anxiety state, targeting processing and attention orienting [26].

Investigations in adults have established a correlation between migraine and internalizing psychiatric comorbidities [27]. Evaluated by emotional rating scales, although no discrepancy was obtained in SDS analysis, migraineurs reported higher scores on SAS, which was consistent with a previous study using same measurement scales [28]. More important was the phenomenon that female patients were more anxious than male patients, while this sex-related difference did not exist in healthy participants. Similarly, Juang et al. discovered that psychiatric disorders were more frequent in female patients with transformed migraine in comparison with male counterparts [29], and Wilcox et al. also reported a significant female predominance in comorbid anxiety in a recent cohort of 187 adolescents suffering from migraine [30]. Since anxiety and other mood disorders have been shown to be related to higher healthcare expenditures [31], poor quality of life [32] and even increased suicidal risk for migraineurs [33], as well as influencing their symptomology, clinical course and treatment response [34], it is reasonable to conclude that psychiatric comorbidities could lead to increased burdens as mentioned above, and to some extent, affecting clinical outcomes of migraine patients, such as perceived disability [35] and migraine chronification [36], particularly for women who seemed to have higher levels of anxiety.

P3, including P3b and P3a, is a generic name of various relatively late and positive components [21]. This reliable component is considered as mirroring aspects of cognitive processing, with its amplitude widely used as a measure of processing capacity and largely determined by attention allocation, working memory and decision making [37]. In this study, we discovered significant group effects in amplitudes of P3 and difference P3 components, indicating impaired cognitive processing of visual information in migraineurs overall. Consistently, other researchers found reduced P3 amplitude in migraine patients as well [38, 39]. Following analysis revealed significant difference between female patients and female controls, but not for male subjects. Interestingly, compared with male patients, lower amplitude was observed in female patients after controlling for age, education and other clinical characteristics. This disease-specific finding was further validated in P3d_T_ and P3d_N_ components, which are believed to better represent attention and response towards task-relevant and deviant stimuli. The aforementioned results suggested that female patients might suffer from more severe abnormalities in visual neurocognitive processing under attentional conditions, such as target processing and attention orienting, at least in the interictal period.

P2, a cognitive-evaluative component with a predominant frontal distribution at around 200 ms post-stimulus, is indicative of early perceptual processing, feature detection, automatic evaluation and attentional recruitment [40, 41]. Considering that the frontal region is crucial for these processes, P2 component could be employed to reflect frontal activity. Nevertheless, we failed to observe conspicuous P2d_T_ due to a subset of participants displaying a difference “negativity”, regardless of groups or genders. This subset of subjects did not differ in any characteristics from the other tested subjects. Although group effect was not found for P2 in original waveforms, it existed in difference P2d_N_ amplitude, implying potential frontal dysfunction in migraine sufferers, which was verified in previous researches [7, 42]. In addition, similar sex discrepancies resembling P3 were also obtained in patients, suggesting that female migraineurs might have more serious disturbances in allocating attentional resources for the later cognitive processing than males did.

There are some pieces of indirect evidence supporting our observations. It was demonstrated that female patients suffering from chronic migraine showed more dysfunctional organization in resting functional networks than in male patients [17], and resting networks could be conceived as neurocognitive entities that incorporate both local and global processes [43]. Furthermore, Kruit et al. reported that women, but not men, with migraine with and without aura were at increased risk of high deep white matter lesion load [44]. Additionally, Palm-Meinders et al. discovered that female sufferers had a higher prevalence and a greater increase of deep white matter hyperintensities at the 9-year follow up [45], and white matter hyperintensities as well as other structural abnormalities have been linked to multi-domain cognitive impairment in migraineurs and the elderly [9, 46]. However, larger amplitudes of N2 and N2d (target minus standard) were obtained in male patients than female patients using an auditory oddball paradigm [38], which were not found in our study. The inconsistencies might arise from different stimuli used for ERP elicitation and distinct criteria for participant selection. So different paradigms and standardized experimental procedures should be used in future studies. More importantly, since migraine is known to be a multifactorial disorder, it is possible that the observed sex differences in cognitive processing might be affected by various determinants, including sex hormone fluctuations and genetic variance [11, 16], while the exact mechanisms deserve further elucidation.

To the best of our knowledge, this was the first study to report gender-related differences in neurocognitive processing of visual stimuli and topographical voltage distributions for migraineurs. A sensitive three-stimulus paradigm and difference waveforms were employed to make the results more convincing. Besides, sex discrepancies in emotional characteristics were investigated, and the effect of emotion on cognitive ERPs was almost ruled out (Pearson’s correlations, see Additional file 1: Table S1). However, our study had several limitations when interpreting the present findings. Firstly, the small cohort did not include migraine patients with aura, which needed further investigation. Moreover, we recruited participants with broader age range, thus making the data less homogenate and resulting in lower statistical power. Finally, source analysis was not performed in this experiment, which we wished to work on in the near future to reveal neural generators of corresponding ERP components, and more microscopically, uncover possible gender-specific differences at the network level.

## Conclusions

The present study revealed gender-related differences in interictal migraine patients without aura. We observed that the emotion and attentive visual processing of females seemed to be more vulnerable to migraine, for example, higher anxiety levels and lower amplitudes of cognitive original/difference ERP components. Our findings suggest the existence of gender effect and the importance of considering gender when investigating neurocognitive processing of migraineurs. In addition, it was demonstrated that migraine patients overall suffered from attentive processing abnormalities, which showed a skewed balance between males and females. Given this, in order to facilitate better outcomes for patients, it is important to highlight cognitive-behavioral therapy, and sex should also be taken into account, especially for women whose abnormalities tended to be more severe, at least indicated by this study. Moreover, in order to better understand gender-related discrepancies of migraine and their diverse mechanisms, more investigations are still warranted. Cognitive ERP components, such as P3, might serve as promising electrophysiological biomarkers to investigate gender-related differences of migraine in specific domains, while the availability needs to be further validated.

## Additional file

Additional file 1: **Table S1.** Correlations between ERP data and emotional characteristics in migraineurs. (DOCX 19 kb)

## Abbreviations

ANOVA: Analysis of variance
EEG: Electroencephalogram
EOG: Electrooculogram
ERP: Event-related potential
ICA: Independent component analysis
ICHD-3 beta: Beta version of the International Classification of Headache Disorders, 3rd edition
P2d_N_: P2 novel effect (novel minus standard)
P2d_T_: P2 target effect (target minus standard)
P3d_N_: P3 novel effect (novel minus standard)
P3d_T_: P3 target effect (target minus standard)
SAS: Self-Rating Anxiety Scale
SD: Standard deviation
SDS: Self-Rating Depression Scale
VAS: Visual analog scale
*η*^2^: Partial eta squared
