# Supplementary material for: Female versus male migraine: an event-related potential study of visual neurocognitive processing
Source: J Headache Pain. 2019 Apr 23;20(1):38. doi: 10.1186/s10194-019-0995-y (PMC6734427; doi:10.1186/s10194-019-0995-y)
Supplement: Supplementary file 1 — Table S1. Correlations between ERP data and emotional characteristics in migraineurs. (DOCX 19 kb) [file 10194_2019_995_MOESM1_ESM.docx]

**Table S1** Correlations between ERP data and emotional characteristics in migraineurs

|  | SAS score | SDS score |
| --- | --- | --- |
| Original/difference ERP data | *r* (*p* value) | *r* (*p* value) |
| P3 amplitude | −0.223 (0.136) | −0.174 (0.248) |
| P3 latency | 0.157 (0.299) | −0.017 (0.910) |
| N1 amplitude | −0.030 (0.845) | 0.226 (0.131) |
| N1 latency | 0.029 (0.848) | 0.018 (0.906) |
| P2 amplitude | −0.355 (0.015)* | −0.118 (0.435) |
| P2 latency | −0.088 (0.560) | 0.024 (0.873) |
| N2 amplitude | −0.030 (0.844) | 0.104 (0.493) |
| N2 latency | 0.063 (0.677) | 0.097 (0.522) |
| P3d_T_ amplitude | −0.041 (0.785) | −0.010 (0.947) |
| P3d_T_ latency | 0.08 (0.595) | 0.026 (0.864) |
| P3d_N_ amplitude | −0.205 (0.172) | 0.004 (0.980) |
| P3d_N_ latency | 0.267 (0.072) | 0.019 (0.899) |
| P2d_N_ amplitude | −0.223 (0.136) | −0.174 (0.248) |
| P2d_N_ latency | 0.126 (0.404) | 0.031 (0.839) |

*r* represents Pearson product-moment correlation coefficient.

SAS: Self-Rating Anxiety Scale; SDS: Self-Rating Depression Scale.

P3d_T_, P3d_N_ and P2d_N_ represent the P3 target effect (target minus standard), P3 and P2 novel effects (novel minus standard), respectively.

**P* < 0.05 by Pearson’s correlations (two-tailed).
